# Implementation of 4M Age-Friendly Care in Arkansas Rural Primary Care Clinics Led to Improved Advance Care Planning

**DOI:** 10.1093/geroni/igab046.3016

**Published:** 2021-12-17

**Authors:** Leah Tobey, Robin McAtee

**Affiliations:** University of Arkansas for Medical Sciences, Little Rock, Arkansas, United States

## Abstract

The 4Ms Age-Friendly Framework has been introduced and implemented into nearly 2,000 primary care practices across the United States by Geriatric Workforce Enhancement Program’s (GWEP) educational efforts. The AR Geriatric Education Collaborative, the GWEP in Arkansas, has provided monthly trainings to a rural federally qualified healthcare clinic system and educated clinicians about how to complete a Medicare Annual Wellness Visit (AWV) that was inclusive of an advance care plan. Specific educational training including the two main components of an ACP: living will preferences and medical power of attorney were reviewed as their role into “What Matters” was explained. Before 4Ms Age Friendly training, baseline data showed only 7% of older adults (OAs) had an established ACP in site 1 and 33% in site 2. After training, the rate of ACP rose to 47% in site 1 and 59% in site 2. During the training, not only were the two main components reviewed but case studies were provided about what questions to ask surrounding the “What Matters” question as a guide to further discuss an OAs wishes, priorities and end-of-life care. This project demonstrated that implementation of 4Ms Age-Friendly Care not only improves the completion of advance care plans but also further enhances the overall care of the older adult when “what matters” most to the older adults is known and communicated.

